# Data Compression by Shape Compensation for Mobile Video Sensors

**DOI:** 10.3390/s90402461

**Published:** 2009-04-09

**Authors:** Ben-Shung Chow

**Affiliations:** Department of Electrical Engineering, National Sun Yat-Sen University, Kaohsiung, Taiwan, 80424, ROC; E-Mail: bschow@mail.ee.nsysu.edu.tw; Tel. +886-07-525-2000 (Ext. 4172);Fax: +886-07-525-4199

**Keywords:** Camera sensor, Low resolution, Mobile video

## Abstract

Most security systems, with their transmission bandwidth and computing power both being sufficient, emphasize their automatic recognition techniques. However, in some situations such as baby monitors and intruder avoidance by mobile sensors, the decision function sometimes can be shifted to the concerned human to reduce the transmission and computation cost. We therefore propose a binary video compression method in low resolution to achieve a low cost mobile video communication for inexpensive camera sensors. Shape compensation as proposed in this communication successfully replaces the standard Discrete Cosine Transformation (DCT) after motion compensation.

## 1. Introduction

Monitoring the environmental conditions of places like museums, buildings, or archaeological sites requires wide nets of different sensing devices [1]. Likewise, there should be different visual quality requirements for video sensor applications. Most security systems, with their transmission bandwidth and computing power both being sufficient, emphasize their automatic recognition techniques. In some situations such as baby monitors and intruder avoidance, the decision function of security systems can be shifted to the concerned human if the video information can be provided inexpensively. For instance, the mother does not need to know the details of her baby’s face, but the general behavior of her baby, like whether it has fallen down from the bed. With the possibility of very small displays on wrist-watch type cellphones, the low visual quality is tolerated with the convenient help of human decision-making. Therefore, the promotion of mobile video communication under very low data rate such as below 10 Kbps is appropriate now.

Video coding with a bit rate below 10 Kbps has not been considered as a practical application in commercial communication systems because speech transmission is conventionally the major application for the real time communication. As a result, a user in the present multiple access system is usually assigned a fixed conceptual channel with the bit rate of speech signal. For example, the basic rate in ISDN (Integrated Services Digital Network) was 2B + D = 2 × 64 + 16 Kbps in earlier days. However, packet transmission, which did not exist in 2G or older systems, has emerged in 2.5G and 3G systems. This tendency implies wireless telecommunication can be charged according to the amount of data packets transmitted, therefore there is a strong incentive to develop a very low-bit rate video coding from a new perspective. That is, instead of using the expensive compression technique such as model based methods [2], a binary low resolution video compression method with readable and convenient features and low transmission costs is proposed.

Wireless transmission sometimes consumes significantly more power, compared with internal computation [3,4]. In this sense, very low bit-rate data compression, which implies less transmission, is desirable for reducing the battery consumption to gain longer operation time for the battery-powered sensors. However, the MPEG/H.26x series are still the mainstream for sensor applications according to the literature [5,6]. One exception is artificial retina in large scale integration [7], in which the stream used has a resolution of 32 × 32 pixels without any compression. This proposed compression is believed by us the first research on low resolution binary images or videos.

## 2. Shape Compensation

**Figure 1.**
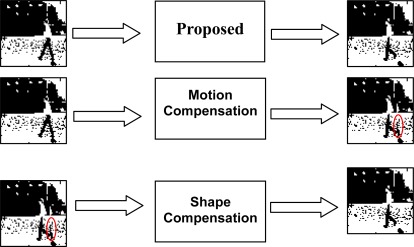
A schematic diagram to present the idea of shape compensation.

The standard dynamic image compression is usually composed of motion compensation and a DCT residue compression. Motion compensation is efficient for binary mode [8]. However, the DCT coding, due to its broad dynamic range in binary mode, would function as a data expansion for the binary images. The binary images are usually described properly by their shapes. In this sense, a novel idea of shape compensation is proposed to replace the DCT. A schematic diagram to present this idea is illustrated in Figure 1.

More clearly, our binary images are coded by the motion vectors and the kinds of shape transformations. For this transformation, a morphological filter is selected to modify the shape of the objects in the image. The morphology processing treats the image components as sets and deals with the changes of shapes very efficiently [9]. Thus, the morphology processing has recently been applied successfully in the auto-inspection and medical image processing industries, but it has not been applied to compression except for the preprocessing for simplifying images [10].

## 3. Selection of Morphological Filters: On-line Selection

In the encoding stage, every motion compensated block has a shape compensation by a suitable morphological filter. This filter is selected on-line from a set of filters, which is selected off-line based on known statistics and experiences. The selection is by voting strategy. The off-line selection will be explained in the next section. We will focus on on-line selection in this section.

The concept of shape compensation is implemented on two image blocks: source block and target block. In the proposed compression method, the source block is the motion compensated previous coded block and the target block is the current coding block. The source block is shape compensated (filtered) to look like the target block. The frames are further divided into blocks of 16 × 16 pixel size. We then define the pattern with size of 3 × 3 with its center as the working pixel on the source block. Thus, there are 16 × 16 moving patterns in each scanned block. Patterns of the same kind are grouped together as pattern group. Therefore there are at most 512 pattern groups. We need to select a filter for each source block. The filters for selection are candidates. Every pattern in the block is a voter and a pattern group is a voter group. Thus, there are 16 × 16 voters of at most 512 voter types for every block. We can prepare the filter-pattern relation table off-line by filtering every type of pattern. One typical table composed of 256 filters is shown in Table 1.

We then define the target pixel on the target block by the corresponding pixel with the same location of the center of the pattern (voter) on the source block. For each pattern (voter), the filtered result is either consistent with the target pixel or not. The optimal filter is the filter causing the least inconsistent results. In other words, the candidate on selection is the candidate accepted by the most voters. The optimal filter (winner) can be selected pattern (voter) by pattern (voter) or group by group. Group is short for pattern (voter) group. Selection by group is most advantageous in the situation where the voters are much more than the groups and is occurred during the off-line selection in the next section. The pattern group associated with the target value is called pattern-target relation or pattern-target occurrence table. One realization of this relation is shown in Table 2.

In practice, we first scan the source and target blocks to have Table 2 (pattern-target occurrence table). Then, we build a pattern-filter conflict table from Table 1 (the off-line prepared pattern-filter relation table) and Table 2 (pattern-target occurrence table). Using this table, the least inconsistent filter is obtained. One example of the pattern-filter conflict table deduced from Table 1 and Table 2 is shown in Table 3.

We summarize the processing procedures as follows:

Step 0. Off-line preparing the filter collection (explained in next section) and Table 1 (Filter-pattern relation) table.
Step 1. Building Table 2 (Pattern-target occurrence table) in a single scan of the corresponding source and target blocks.
Step 2. Building Table 3 (Filter-pattern conflict table) from Table 1 and Table 2 by checking the target value in Table 2 with the relation value in Table 1.
Step 3. Finding the least conflicts filter from Table 3 by summing the column.

## 4. Reduction of Morphological Filters: Off-Line Selection

The candidate filter must be selected first before on-line application because there are too many theoretically possible morphological filters from Matheron representation theorem [9]. For example, the number of decreasing morphological filters composed of two structuring elements (mask) with size of 3 × 3 is 11645 computed below:

(1) $$(\sum_{i=1}^{9}C_{i}^{9}(2^{9}-2^{i}-2^{9-i}+1)\;)/2$$

where i is the number of 1’s in the 3 × 3 mask.

**Figure 2.**
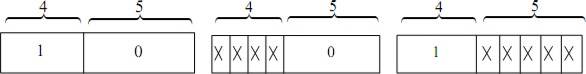
Illustrations for 3 × 3 mask expressed by 9 × 1 mask to explain the relation between masks. (a) One special mask A (working as the first mask) (b) The mask prototype for masks contained in mask A. (c) The mask prototype for masks containing mask A.

We can understand Equation (1) by a special case when *i* = 4. For this case, there are $C_{4}^{9}$ possible variations for the first mask. It should be noted that two masks must be not included to each other to avoid redundant computation. To chose the second mask for a special mask A (working as the first mask) in Figure 2(a) from the mask set (with size of 2^9^), any mask in the form of Figure 2(b) (with 2^4^ variations) or Figure 2(c) (with 2^5^ variations) cannot be selected because they must be contained or contain mask A. One should be added up in Equation (1) because the mask set in Figure 2(b) and the mask set in Figure 2(c) have one common mask, which is mask A. Finally, the total number should be divided by 2 since there is no order difference for the first and second mask.

**Figure 3.**
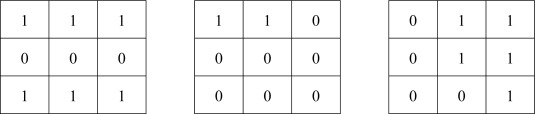
Examples of mask for opening or closing.

We select the most preferable 100 filters from 11,645 filters according to six different streams of 60 frames (6 × 60 × 64 × 64 pixels in total) using the same methodology used in the on-line selection explained in the previous section. Similarly, we select another 100 filters for increasing morphological filters. Furthermore, we add in extra 56 opening and closing filters composed of one mask only, based on our experience, to make a total of 256 candidates. Examples of mask for opening or closing are shown in Figure 3.

## 5. Experimental Results

The video streams of a walking girl were tested in our experiment. They were first reduced to a 64 × 64 pixel size and then thresholded to be bi-level 64 × 64 sequences. Binary motion search and morphological filter determination was performed at the encoding stage. The block would be refreshed if the motion compensated error were above the threshold. Correspondingly, there are two possibilities in the decoding process: block refreshing or shape compensation after motion compensation.

**Figure 4.**
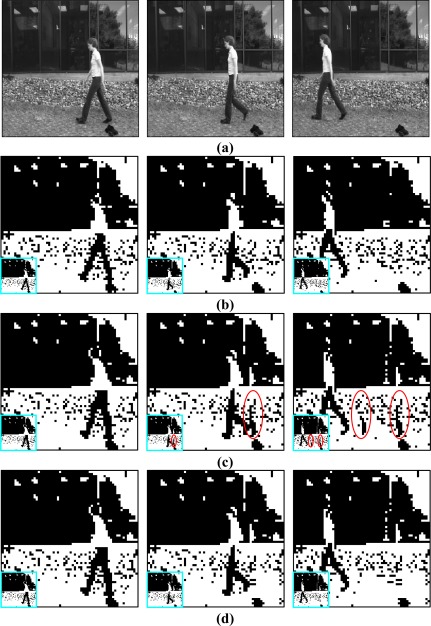
Illustrations for some sampled frames from walking girl video streams. (a) Top row: Original frames in the grey form with size of 256 × 256. (b) The second row: Original frames in the binary form with size of 64 × 64. (c) The third row: Motion compensated frames in the binary form with size of 64 × 64. (d) Bottom row: Shape compensated frames in the binary form with size of 64 × 64. Left column: Frame 6. Middle column: Frame 12. Right column: Frame 45. All binary images are magnified to see the details, also with the non-magnified images located in the left bottom for visual comparison.

Some sampled frames are selected in Figure 4 to demonstrate the effect of shape compensation. The original grey 256 × 256 pixels and bi-leveled 64 × 64 pixels are also shown in Figures 4(a) and 4(b) for reference. The magnified 64 × 64 images are also shown in Figure 4 for the details. The motion compensation flaws are circled to stress the shape compensation requirement. It is noted that the flaws circled in Figure 4(c) are fixed by shape compensation without circles in Figure 4(d), which appear similar to the original images in Figure 4(b). The flows of motion compensation are caused by a faulty motion vector, usually the zero motion. The morphological filter selected to fix the flaw of left over shown in Figure 4(c) is an opening operation with a shape of lower triangle defined in a 3 × 3 mask.

In our experiments, the testing images are of the 64 × 64 pixel size. The block sizes of the motion compensation and shape compensation are both 16 × 16. The range of motion search is also 16 × 16, at the expense of 0.128k bits per frame for coding. The number of kinds of shape transformation is about 256 for two masks, costing around 0.128 k bits per frame for coding the shape transform. The average block (or frame) refreshing ratio is about 0.06. The frame rate of 20 frames per second is a very strict requirement for low bit rate video coding. To summarize, a total data rate of 8 Kbps can be achieved for the testing stream computed by (0.128 + 0.128 + 64 × 64 pixels/frame × 0.12) × 20 frame/second. Compared to the original bit rate, 64 × 64 pixels/frame × 20 frame /second, 80 Kps, the compression ratio of this proposed method is above 10.

**Figure 5.**
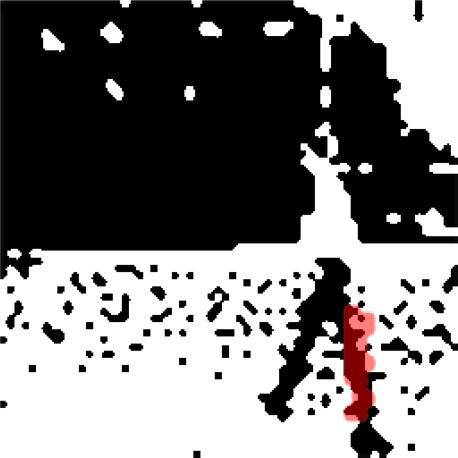
The error improvement is significant (about 50%) if focused on the significant moving area; the average error count decrease from 20.4 to 13.5 for the shaded region with red color for the frames from seven to forty five.

In sensing application, the moving object is usually the important target to sense or even recognize. We therefore specifically compute the error rate for the moving object (with a significant motion), which is important for human’s visual perception. This error rate is also a key factor for reducing false recognition rate when applied to recognition application. For example, motion segmentation and motion region analysis are investigated for object recognition in the literature [11,12]. We therefore compare the error rate for this specific region in Figure 5 for two methods: the proposed method V.S. the method using motion only without shape compensation. The error rate for the whole image is about 5%, which is not much better than motion only method. However, the error improvement by shape compensation is significant (about 50%) if focused on the significant moving area.

JBIG2 is a compression standard for binary images of document storage because it usually takes advantage of the textual and halftone data, and uses the models designed specifically for those data types. Our experiments on JBIG2 with software provided by the manufacturer [13] using a linear interpolation method for the “Walking girl” test image presents a compression ratio only about 2, which is consistent with the literature report [14]. We also find a compression ratio of about 10 for JBIG2 from a literature report [15]. The compression ratio of [15] may be much lower if their testing images, natural images of 512 × 512, is reduced to 64 × 64 because small size natural images are very difficult to compress.

## 6. Conclusions

Our compression method is simplely motion compensation followed by the shape compensation. Shape compensation, replacing the standard DCT due to the broad dynamic range of DCT in binary mode, can usually fix the problems caused by motion compensation as shown in our experiments. This compensation is a new method proposed for sensor applications. Our compressing method is justified by three perspectives: 1. Decision function is fulfilled with the help of users themselves. 2. Thus, the resources (bandwidth and power) are saved. 3. This limited resources approach has not been investigated because of the fix-rate charge policy for telecommunications before our 3G era. This proposed compression is believed by us the first research on binary image or videos in low resolution.

## Figures and Tables

**Table 1.** Filter-pattern relation table.

**Filter - Pattern Relation**				

Filter	Filter 1	Filter 2	…	Filter 256
Pattern				

Pattern Type	Filtered Value	Filtered Value		Filtered Value
Type 1	1	0		0
Type 2	1	1		0
⋮	⋮	⋮		⋮
Type 512	1	0		0

**Table 2.** Pattern-target occurrence table.

**Pattern-Target Occurrence**		
Pattern Type	Target Value	Occurrence #
Type1	0	5
	1	16
Type 2	0	20
	1	4
⋮	⋮	⋮
Type 512	0	20
	1	4

**Table 3.** Pattern-filter conflict table.

**Filter - Pattern Conflict Occurrence**				

Filter	Filter 1	Filter 2	…	Filter 256
Pattern				

Pattern Type	Occurrence #	Occurrence #		Occurrence #
Type 1	5	16		16
Type 2	20	20		4
⋮	⋮	⋮		⋮
Type 512	20	4		4
Total Conflict	127	204		123
